# Life events and psychosis: case–control study from India, Nigeria, and Trinidad and Tobago

**DOI:** 10.1192/bjo.2022.562

**Published:** 2022-09-16

**Authors:** Ibidunni O. Oloniniyi, Helen A. Weiss, Sujit John, Oluyomi Esan, Maia Hibben, Vikram Patel, Robin M. Murray, Alex Cohen, Gerard Hutchinson, Oye Gureje, Rangaswamy Thara, Craig Morgan, Tessa Roberts

**Affiliations:** Department of Health Services and Population Research, Institute of Psychiatry, Psychology and Neuroscience, King's College London, UK; MRC International Statistics and Epidemiology Group, London School of Hygiene & Tropical Medicine, UK; Department of Psychiatry, Schizophrenia Research Foundation, India; Department of Psychiatry, University of Ibadan, Nigeria; Department of Psychiatry, Faculty of Medical Sciences, University of the West Indies, Trinidad and Tobago; Department of Global Health and Social Medicine, Harvard Medical School, Massachusetts, USA; and Department of Global Health and Population, Harvard T.H. Chan School of Public Health, Harvard University, Massachusetts, USA; Department of Psychosis Studies, Institute of Psychiatry, Psychology and Neuroscience, King's College London, UK; Centre for Global Mental Health, London School of Hygiene & Tropical Medicine, UK; WHO Collaborating Centre for Research and Training in Mental Health, Neuroscience and Substance Abuse, Department of Psychiatry, University of Ibadan, Nigeria; ESRC Centre for Society and Mental Health, King's College London, UK

**Keywords:** Psychosis, life events, India, Nigeria, Trinidad and Tobago

## Abstract

**Background:**

There is evidence of an association between life events and psychosis in Europe, North America and Australasia, but few studies have examined this association in the rest of the world.

**Aims:**

To test the association between exposure to life events and psychosis in catchment areas in India, Nigeria, and Trinidad and Tobago.

**Method:**

We conducted a population-based, matched case–control study of 194 participants in India, Nigeria, and Trinidad and Tobago. Cases were recruited through comprehensive population-based, case-finding strategies. The Harvard Trauma Questionnaire was used to measure life events. The Screening Schedule for Psychosis was used to screen for psychotic symptoms. The association between psychosis and having experienced life events (experienced or witnessed) was estimated by conditional logistic regression.

**Results:**

There was no overall evidence of an association between psychosis and having experienced or witnessed life events (adjusted odds ratio 1.19, 95% CI 0.62–2.28). We found evidence of effect modification by site (*P* = 0.002), with stronger evidence of an association in India (adjusted odds ratio 1.56, 95% CI 1.03–2.34), inconclusive evidence in Nigeria (adjusted odds ratio 1.17, 95% CI 0.95–1.45) and evidence of an inverse association in Trinidad and Tobago (adjusted odds ratio 0.66, 95% CI 0.44–0.97).

**Conclusions:**

This study found no overall evidence of an association between witnessing or experiencing life events and psychotic disorder across three culturally and economically diverse countries. There was preliminary evidence that the association varies between settings.

Life events have been defined as discrete or significant experiences that disrupt the usual activities of an individual, resulting in a substantial change and readjustment.^1^ Such events can be considered as stressors, and have been shown to have important effects on health and psychological well-being.^2^ The effect of life events depends partly on their nature, with undesirable, unpredictable or uncontrollable events more likely to have negative consequences for mental health than events without these characteristics.^3^ The impact of life events may also depend on the subjective significance given to these events by the individuals who experienced them.^4^

Life events have been shown to affect the onset, course and outcome of psychotic disorders.^5^ The biological mechanism through which life events affect psychosis remains unclear,^6^ although various models have been postulated.^7,8^ Events throughout the life course, including adverse childhood experiences, have been hypothesised to increase predisposition to psychosis,^9^ whereas recent events occurring shortly before the onset of psychosis may trigger the onset of symptoms in those who are already vulnerable.^10^

## Associations between life events and psychosis

A meta-analysis of the association between childhood adverse experiences and psychosis showed a three-fold increase in the odds of psychosis among people who suffered childhood trauma compared with people without such experiences.^11^ The majority of studies that have investigated the associations between recent life events and psychosis have also observed an increase in life events before the onset of psychosis.^1^ However, almost all of these studies were conducted in Western Europe, North America and Australasia, so the extent to which the associations hold in other populations is unknown. Since the effect of life events is dependent on the nature of the events and their significance to those who experienced them, the experience of life events may differ between settings not only in terms of their types and frequency, but also in how they are interpreted and responded to. Further, the impact of life events may be buffered by social support,^12^ which varies both in nature and extent between contexts.^13^ Thus, caution must be exercised in generalising findings from Western Europe, North America and Australasia to the rest of the world.

Few studies have investigated the associations between life events and psychosis in the Global South. In Nigeria, Gureje and Adewunmi^14^ compared 42 people with a diagnosis of schizophrenia, at their first episode, with 57 controls, and found that the onset of illness among cases was not preceded by an increase in life events relative to controls. In India, Chakraborty et al^15^ compared life events (lifetime, 6 months, 3 months and 2 weeks) occurring in 18 participants before the onset of acute and transient psychotic disorder, and manic episodes occurring in 20 participants. They observed a higher frequency of impersonal events, undesirable events and higher stress scores among those with acute and transient psychotic disorder compared with those with mania without psychosis.^15^ In a Tanzanian study where 899 community members were studied, individuals who had experienced two or more recent life events in the previous 6 months had higher odds of experiencing psychotic symptoms than those who experienced one or fewer life events.^16^ All of these studies explored life events within 6 months preceding onset of psychosis (i.e. recent events that may have triggered psychosis) rather than events occurring across the lifetime (i.e. events increasing vulnerability to psychosis).

## Current study

This present study addresses an evidence gap of the possible impact of life events (not restricted to recent events) on the causes of psychosis in more diverse settings.

The objective of this exploratory analysis of data from a pilot study in India, Nigeria, and Trinidad and Tobago is to assess the association between witnessing or experiencing life events and psychotic disorder (both affective and non-affective). This will generate preliminary findings to inform future studies in these countries.

## Method

### Study design

This is a secondary analysis of data from a case–control study entitled International Research Programme on Psychoses in Diverse Settings I (INTREPID I);^17^ data were collected in 2014. INTREPID I was a population-based programme designed to implement and evaluate methods of identifying, assessing and following untreated cases of psychosis (affective and non-affective psychosis) and matched controls in catchment areas of India, Nigeria, and Trinidad and Tobago.^17,18^ It was a pilot study to inform subsequent larger-scale studies.

### Setting

Data were collected from catchment areas in three sites: Chengalpet taluk (near Chennai), Tamil Nadu, India (population 412 289);^19^ Ibadan South East and Ona Ara Ibadan, Oyo-State, Nigeria (population 532 032);^20^ and Tunapuna-Piarco, Trinidad, Trinidad and Tobago (population 248 656).^21^

In India, 32.7% live below the poverty line ($1.25 per day)^22^ and 74% are literate,^22^ according to 2010 figures. In Nigeria, 68.2% live in poverty^22^ and 60% are literate.^23^ In Trinidad and Tobago, 4% live in poverty^22^ and 99% are literate.^24^ Detailed socioeconomic data are not available at the level of the catchment areas, but more details about the health systems in the catchment areas have been published previously.^17^

### Participant recruitment and sample size

Details on recruitment of cases and controls have been reported previously.^17^ Brief details are provided below.

### Cases sampling and recruitments

Mapping exercises of service providers and investigations of help-seeking patterns in each site established comprehensive, bespoke case detection systems, which incorporated all public and private professional mental health providers, the major spiritual and traditional healers, and a network of key informants.^17^ These networks are regularly monitored by the research teams in each site to identify potential cases, who were then screened with the Screening Schedule for Psychosis,^25^ and those who screened positive were invited to participate.

### Inclusion criteria for cases

Inclusion criteria were as follows: age 18–64 years, resident in the catchment area at the time of case detection, meet the ICD-10 diagnostic criteria for psychotic disorder in past 12 months and not treated with antipsychotics for 3 continuous months before the start of recruitment.

### Exclusion criteria for cases

Exclusion criteria were as follows: evidence of psychotic symptoms precipitated by an organic cause, central nervous system disease and transient psychotic symptoms resulting from acute intoxication.

### Control sampling and recruitments

Controls were individually matched to cases by gender, age and neighbourhood; Trinidad and Tobago also matched by ethnicity. To identify controls, the ten most proximal households to where each case lived were identified, and each of these ten households were then contacted. The age and gender of each resident was recorded, and all gender- and age- (within 5 years of the case) matched residents were listed in random order and approached consecutively until a suitable control was recruited. If a suitable control was not recruited from the list of ten households, the process was repeated with the next ten households.

### Inclusion criteria for controls

Inclusion criteria were as follows: residents in catchment areas, aged 18–64 years, within 5 years of age of the matched case, same gender as matched case, screened negative for psychosis with the Screening Schedule for Psychosis^25^ not related to the cases and consented to participate.

### Data collection

Data were collected by trained research workers via face-to-face interviews.

### Measures

The Screening Schedule for Psychosis^25^ was used to screen all potential cases and controls to determine eligibility. This has been widely used to screen for psychosis across settings.

The Schedule for Clinical Assessment in Neuropsychiatry^26^ interview was used to confirm diagnosis in cases and was administered by experienced clinicians in each site.

The Medical Research Council Sociodemographic Schedule^27^ was used to collect information about gender, age, education level, employment status, income, housing and relationship status. Information regarding income was obtained in the local currency of each country, which was then grouped by centiles within each site into ‘high’, ‘middle’ and ‘low’ to relativise them to the setting; these values were then converted to dollars, using the exchange rate for the year of the study.^28–30^ Educational level was operationalised based on the number of years spent schooling, and then grouped.

The Global Assessment of Functioning^31^ is a numeric scale used to rate individuals’ social, occupational, and psychological functioning. The split version with separate scales for symptoms and functioning/disability was used.^32^

The Harvard Trauma Questionnaire (HTQ) (Supplementary Material available at https://doi.org/10.1192/bjo.2022.562) was used to assess lifetime occurrence of traumatic life events among study participants.^33^ It contained a list of 17 traumatic events with options to mark as ‘no event’, ‘witnessed’ and ‘experienced’. Traumatic events were recorded as ‘no event’ (0) and ‘witnesses or experienced’ (1). The time period when the life event occurred was not indicated.

### Statistical analysis

A descriptive exploration of the data was conducted by examining the variables range, mean and s.d. (for normally distributed continuous variables), median and interquartile range (for continuous variables with skewed distributions), percentages (for categorical variables) and missing values. Histograms were plotted to assess the distributions of the continuous variables.

Cross-tabulations were used to summarise sociodemographic characteristics, clinical characteristics and exposure to life events by case–control status. Fishers exact test was used when cells contained five or fewer participants. Life events were the main exposure of interest and were treated as a continuous variable. Individuals who reported having either experienced or witnessed a life event were categorised as exposed.^33^

As cases and controls were individually matched on age group, gender and neighbourhood, a matched analysis was conducted, using conditional logistic regression to estimate odds ratios and 95% confidence intervals. We first performed univariable analysis, and then proceeded to multivariable analysis, adjusting for potential confounders (age and educational level). Even though participants had been matched by age group (within 5 years), we still adjusted for age since it was not an exact age match. Sensitivity analyses were conducted with unconditional logistic regression, adjusting for matching factors. The effect of each potential confounder was explored individually on the association between life events and psychosis, and those that changed the odds ratio by 10% or more were included sequentially into the model. The final model was chosen after assessing the fit of the models with likelihood-ratio tests. Since a matched analysis was carried out, only matched pairs contributed to the analysis; as such, five participants with missing items or unmatched sets were dropped from the analysis.

The data were analysed with statistical software Stata version 15.1 for Windows.

### Ethics

The authors assert that all procedures contributing to this work comply with the ethical standards of the relevant national and institutional committees on human experimentation and with the Helsinki Declaration of 1975, as revised in 2008. All procedures involving human patients were approved by the Psychiatry, Nursing and Midwifery Research Ethics Subcommittees of King's College London (approval number PNM1213 119), the Institutional Ethics Committee of the Schizophrenia Research Foundation in India, the University of Ibadan Ethics Committee in Nigeria and the University of the West Indies St Augustine Campus Ethics Committee in Trinidad and Tobago, and permission was also granted by all the relevant local health authorities for the parent study.

Ethical approval for this secondary data analysis was obtained from the London School of Hygiene and Tropical Medicine MSc Research Ethics Committee (approval number 17044).

All participants were informed about the study, that the data would be anonymised and that they were free to withdraw from the study at any time. A written information sheet in the local language was provided to the participants; this was signed by all literate participants and read out to non-literate participants in the presence of a witness, to obtain recorded verbal informed consent.

## Results

### Sociodemographic characteristics of the study population

Overall, 97 cases and 97 controls were included in the study. The mean age of cases was 35 years (s.d. 10.8) and the majority (61.5%) were female. There was evidence that cases had lower levels of education, were less likely to be employed and were more likely to be living with their parents (Table 1).

**Table 1:** Sociodemographic and clinical characteristics of cases and controls

Variables	Cases, *n* = 97, *n* (%)	Controls, *n* = 97, *n* (%)	*P*-value
Age group, years			
18–26	24 (25)	23 (24.7)	0.97
27–35	32 (33.3)	31 (32)	
36–44	19 (19.8)	22 (22.7)	
45–62	21 (21.9)	20 (20.6)	
Mean (s.d.)	35.1 (10.8)	34.8 (11.2)	0.84
Gender			
Male	37 (38.5)	37 (38.1)	0.95
Female	59 (61.5)	60 (61.9)	
Site			
India	44 (45.4)	44 (45.4)	1.00
Nigeria	35 (36.1)	35 (36.1)	
Trinidad and Tobago	18 (18.5)	18 (18.5)	
Educational level			
Primary	34 (41)	18 (20.9)	0.02
Secondary	38 (45.8)	49 (57)	
Tertiary	11 (13.3)	19 (22.1)	
Current employment status			
Unemployed	57 (62.6)	18 (18.6)	<0.001
Employed	34 (37.4)	79 (81.4)	
Monthly income in USD,^a^ median (IQR)	32.7 (147.5)	131.1 (274.8)	<0.0001
Current relationship status			
Single	38 (39.6)	18 (18.6)	<0.001
Married/in a steady relationship	33 (34.4)	76 (78.4)	
Divorced/separated/widowed	24 (25)	3 (3)	
Who do you live with?			
Alone/with children	20 (20.8)	8 (8.3)	<0.0001
Partner/spouse/with children	29 (30.2)	70 (72.9)	
Parents/living with others	47 (49)	18 (18.8)	
GAF disability score, median (IQR)	55 (29)	95 (14)	<0.0001
Number of life events (witnessed or experienced)			
None	36 (38.3)	43 (44.3)	0.39
One or more	58 (61.7)	54 (55.7)	
Median (IQR)	2 (4)	1 (3)	0.25

USD, United States Dollars; IQR, interquartile range; GAF, Global Assessment of Functioning.

a. 2014 currency rate 1USD = 61.00759 Indian Rupees,^34^ 1USD = 156.483 Nigerian Naira^35^ and 1USD = 6.3613 Trinidad and Tobago Dollars.^36^

Among the cases, 61.7% reported witnessing or experiencing lifetime traumatic events, compared with 55.7% of the controls experiencing at least one life event during their lifetime (*P* = 0.39; Table 2).

**Table 2:** Lifetime exposure to life events among participants in India, Nigeria, and Trinidad and Tobago

	India			Nigeria			Trinidad and Tobago		
Life events	Cases, *n* = 44, *n* (%)	Controls, *n* = 44, *n* (%)	*P*-value	Cases, *n* = 35, *n* (%)	Controls, *n* = 35, *n* (%)	*P*-value	Cases, *n* = 18, *n* (%)	Controls, *n* = 18, *n* (%)	*P*-value
Any life events									
No	21 (48.8)	30 (68.2)	0.07	8 (23.5)	11 (31.4)	0.46	7 (41.2)	2 (11.1)	0.04
Yes	22 (51.2)	14 (31.8)		26 (76.5)	24 (68.6)		10 (58.8)	16 (88.9)	
Combat situation									
No	44 (100.0)	43 (100.0)		29 (82.9)	34 (97.1)	0.05^a^	16 (88.9)	12 (66.7)	0.11^a^
Yes	0 (0.0)	0 (0.0)		6 (17.1)	1 (2.9)		2 (11.1)	6 (33.3)	
Lack of food and water									
No	31 (72.1)	39 (88.6)	0.05	13 (37.1)	15 (42.8)	0.63	15 (83.3)	15 (83.3)	0.67^a^
Yes	12 (27.9)	5 (11.4)		22 (62.9)	20 (57.1)		3 (16.7)	3 (16.7)	
Lack of shelter									
No	36 (83.7)	42 (95.5)	0.09^a^	26 (74.3)	26 (74.3)	1.00	17 (94.4)	16 (88.9)	1.00^a^
Yes	7 (16.3)	2 (4.5)		9 (25.7)	9 (25.7)		1 (5.6)	2 (11.1)	
Being close to death									
No	42 (97.7)	41 (93.2)	0.62^a^	22 (62.9)	25 (71.4)	0.45	12 (66.7)	10 (55.6)	0.49
Yes	1 (2.3)	3 (6.8)		13 (37.1)	10 (28.6)		6 (33.3)	8 (44.4)	
Illness without access to medical care									
No	41 (95.4)	44 (100.0)	0.24^a^	27 (77.1)	31 (88.6)	0.34^a^	17 (94.4)	18 (100.0)	1.00^a^
Yes	2 (4.6)	0 (0.0)		8 (22.9)	4 (11.4)		1 (5.6)	0 (0.0)	
Forced separation from friends									
No	38 (88.4)	43 (97.7)	0.11^a^	25 (71.4)	26 (74.3)	0.79	16 (94.1)	16 (88.9)	1.00^a^
Yes	5 (11.6)	1 (2.3)		10 (28.6)	9 (25.7)		1 (5.9)	2 (11.1)	
Unnatural death of friend or family									
No	30 (69.8)	37 (84.1)	0.11	18 (51.4)	19 (54.3)	0.81	16 (88.9)	9 (50.0)	0.03
Yes	13 (30.2)	7 (15.9)		17 (48.6)	16 (45.7)		2 (11.1)	9 (50.0)	
Lost or kidnapped									
No	40 (93.0)	43 (97.7)	0.36^a^	28 (80.0)	27 (77.1)	0.77	15 (83.3)	18 (100.0)	0.23^a^
Yes	3 (7.0)	1 (2.3)		7 (20.0)	8 (22.9)		3 (16.7)	0 (0.0)	
Torture									
No	37 (86.0)	43 (97.7)	0.06^a^	21 (60.0)	25 (71.4)	0.31	17 (94.4)	17 (94.4)	1.00^a^
Yes	6 (14.0)	1 (2.3)		14 (40.0)	10 (28.6)		1 (5.6)	1 (5.6)	
Murder of friend or family									
No	43 (100.0)	44 (100.0)		27 (77.1)	31 (88.6)	0.34^a^	15 (83.3)	11 (61.1)	0.26^a^
Yes	0 (0.0)	0 (0.0)		8 (22.9)	4 (11.4)		3 (16.7)	7 (38.9)	
Serious injury									
No	41 (95.4)	43 (97.7)	0.62^a^	28 (80.0)	24 (68.6)	0.27	15 (83.3)	10 (55.6)	0.15^a^
Yes	2 (4.6)	1 (2.3)		7 (20.0)	11 (31.4)		3 (16.7)	8 (44.4)	
Imprisonment									
No	41 (95.4)	44 (100.0)	0.24^a^	25 (71.4)	27 (77.1)	0.58	15 (83.3)	13 (72.2)	0.69^a^
Yes	2 (4.6)	0 (0.0)		10 (28.6)	8 (22.9)		3 (16.7)	5 (27.8)	
Murder of strangers									
No	43 (100.0)	44 (100.0)		33 (94.3)	34 (97.1)	1.00^a^	17 (94.4)	17 (94.4)	1.00^a^
Yes	0 (0.0)	0 (0.0)		2 (5.7)	1 (2.9)		1 (5.6)	1 (5.6)	
Sexual abuse or rape									
No	41 (95.4)	44 (100.0)	0.24^a^	29 (82.9)	32 (91.4)	0.47^a^	15 (83.3)	17 (94.4)	0.60^a^
Yes	2 (4.6)	0 (0.0)		6 (17.1)	3 (8.6)		3 (16.7)	1 (5.6)	
Brainwashing									
No	43 (100.0)	44 (100.0)		32 (91.4)	28 (80.0)	0.31^a^	18 (100.0)	16 (88.9)	0.49^a^
Yes	0 (0.0)	0 (0.0)		3 (8.6)	7 (20.0)		0 (0.0)	2 (11.1)	
Forced isolation									
No	41 (95.4)	44 (100.0)	0.24^a^	24 (70.6)	28 (80.0)	0.36	18 (100.0)	14 (77.8)	0.10^a^
Yes	2 (4.6)	0 (0.0)		10 (29.4)	7 (20.0)		0 (0.0)	4 (22.2)	
Other situation that was very frightening									
No	40 (93.0)	44 (100.0)	0.12^a^	29 (85.3)	33 (94.3)	0.26^a^	17 (94.4)	14 (77.8)	0.34^a^
Yes	3 (7.0)	0 (0.0)		5 (14.7)	2 (5.7)		1 (5.6)	4 (22.2)	

a. Fisher's exact test.

Life events were explored by site. Regarding the total number of life events experienced by participants, in India, 51.2% of cases reported witnessing or experiencing lifetime traumatic events, compared with 31.8% of controls (*P* = 0.07). In Nigeria, 76.5% of cases reported witnessing or experiencing lifetime traumatic events, compared with 68.6% of controls. In Trinidad and Tobago, 58.8% of cases reported witnessing or experiencing lifetime traumatic events, compared with 88.9% of controls (*P* = 0.04).

### Association between life events and psychosis

The crude odds ratio of the association between psychosis and life events witnessed or experienced (as binary) was 1.32 (95% CI 0.72–2.39). Age and education were independently associated with the outcome, and adjustment for age and education yielded an adjusted OR (aOR) of 1.19 (95% CI 0.62–2.28) for the association between psychosis and life events witnessed or experienced. Fitting number of life events as a continuous exposure showed little evidence of extra-linear variability, with an increase in odds of psychosis for every additional life event experienced (aOR 1.07, 95% CI 0.92–1.24).

The sensitivity analysis using unconditional logistic regression showed similar results (aOR for the association of psychosis with binary exposure 1.32, 95% CI 0.71–2.46; aOR for association with every additional life event experienced 1.05, 95% CI 0.95–1.17).

There was evidence of an interaction between life events and country (*P* = 0.002). The aOR for the association between number of life events and psychosis was 1.56 (95% CI 1.03–2.35) for India, 1.17 (95% CI 0.95–1.45) for Nigeria, and 0.66 (95% CI 0.45–0.97) for Trinidad and Tobago (Table 3).

**Table 3:** Site-specific association with number of life events (as a continuous variable)

Site	Adjusted odds ratio^a^	95% CI	*P*-value
India	1.56	1.03–2.35	0.04
Nigeria	1.17	0.95–1.45	0.14
Trinidad and Tobago	0.66	0.45–0.97	0.03

a. Adjusted for age group and education level. Test of homogeneity, *P* = 0.002.

## Discussion

This study found no evidence of an association between increased exposure to life events and psychosis.

However, there was evidence of effect modification by site, with evidence of an association observed in India, no evidence of an association in Nigeria, and evidence that controls were more exposed to life events in Trinidad and Tobago (number of controls with life event divided by the total number of controls *v*. number of cases with life event divided by the total number of controls: 16/18 (88.9%) *v*. 10/18 (55.6%)). The reversal in the association between life events and psychosis in Trinidad and Tobago may be a result of recall bias owing to differences in education and symptom profile. Again, these findings should be treated with caution because of the small numbers in each group. This study also provided very preliminary evidence of increased exposure to specific life events among cases compared with controls.

### Strengths and weaknesses

This was a small pilot study with 194 participants (97 cases and 97 controls), which limited the power of the study. A strength of this study was its use of a representative sample of cases obtained via rigorous, comprehensive case-finding methods, which drew on religious and traditional healing sites and key informants in addition to mental health services, unlike studies that recruit solely from psychiatric facilities,^37,38^ which are unlikely to be representative of people with psychosis in the general population, especially in low-resource settings where many do not present to formal psychiatric services. To our knowledge, this is the first study to examine life events across multiple countries in the Global South.

An important limitation in interpreting the findings is the difficulty of determining the direction of causation, because the timing of life events and psychosis was not indicated in this study, and the median duration of untreated psychosis was 232 weeks, 13 weeks and 38 weeks in India, Nigeria, and Trinidad and Tobago, respectively.^17^ Determining the timing of life events among individuals with and without psychosis will be important in future studies to provide evidence of a causal association, and to achieve a better understanding of the effect of childhood versus recent life events on psychosis.

Some important life events of relevance to people in India, Nigeria, and Trinidad and Tobago may not have been fully captured by the HTQ. For instance, the HTQ does not include job losses, unemployment and relationship challenges. These are common occurrences with major potential ramifications, particularly in low- and middle-income countries with little or no welfare or unemployment benefits. This may also highlight the subjective effect of life events on different individuals; it is theoretically possible that the events included in the questionnaire are not those that are experienced to be most intrusive or traumatic in all cultural settings. Although the HTQ has been widely used and validated in the Global South,^38^ it has been mainly used to assess post-traumatic stress disorder in these settings. It has not been validated in India, Nigeria, or Trinidad and Tobago, but it has been used in diverse populations such as Indochinese,^33^ Iraqi refugees^39^ and refugees from Sub-Saharan Africa to France,^40^ among others. Omission of locally salient life events may have led us to underestimate the prevalence of traumatic life events. The use of a locally generated life event scale may be important to consider in capturing life events of importance in different sites.

It is possible that some variables that we treated as confounders could lie on the causal pathway, such as education; however, there was very minimal change in the results when education was excluded.

Since this study is a case–control study, recall bias cannot be ruled out. However, recent studies suggest that the accuracy of reported life events by people with psychosis is high.^41^ Since data were collected through face-to-face interviews, and some of the items in the HTQ are sensitive, reporting bias is also a possibility, especially if relatives are more likely to be present during interviews with cases than with controls (as is common when conducting research in settings such as rural India). This may have differentially affected data from different sites, given variation in cultural norms.

### Comparison with other studies

This study included more women than men (61.5% *v*. 38.5%). This is in contrast to findings from higher-income countries. where typically there are more men than women with psychotic disorders.^34^ This finding may be directly related to the active case finding method employed in this study,^17^ as well as differences in risk of psychosis among men and women in different populations. Individuals with psychosis did not experience a significantly higher median number of overall life events than controls, and there was no overall evidence of an association between total number of events experienced and odds of psychosis. This association is not in the same direction as most studies conducted in Europe, North America and Australasia,^35,36^ which have found a positive association between exposure to life events and psychosis.

The lack of an association in the current study may be because a majority of participants reported exposure to life events: 58% of cases and 54% of controls. Alternatively, it is possible that protective factors such as social support may be more prevalent in some settings, which cushion the effects of life events. Another possible reason for this lack of association may be the higher percentage of women among our study population. Zarulli et al^42^ found that women were more resilient and survived better even during harsh conditions. A previous Nigerian study of life events among patients with first-episode schizophrenia also found no significant difference in the number of life events between patients and controls 6 months before the first episode.^14^

Some of the life events seemed to be more common among individuals with psychosis than the controls, and others were not, although this must be interpreted with caution because of multiple testing. There was some evidence in this study that lack of food and water, as well as torture in India and combat situation in Nigeria, may be associated with increased odds of psychosis. Some evidence suggests that events that are threatening, intrusive or involve a lack of control (such as physical assaults or invasive operations) specifically increase susceptibility to psychosis.^1^ It is also possible that these events (lack of food and water, torture, combat situations) have an additive effect with current and long-standing social disadvantages. such as unemployment, living alone, being single and lower levels of education.^43^

The HTQ has generally been used to measure exposure to life events among groups who have faced adversity, such as refugees, rather than the general population. These studies typically show a higher prevalence of exposure to traumatic events than observed in our study; for example, a study of Mandaean refugees in Sydney^44^ found that 40%, 69%, 54% and 30% of the participants experienced torture, lack of food and water, illness without access to medical care and lack of shelter, respectively, compared with 23%, 39%, 11% and 18%, respectively, for the participants of this study. More research is needed to assess the extent to which variation in exposure to traumatic life events contributes to variation in the incidence of psychosis across settings.

Because of the small sample size of the current study, it was not possible to investigate interactions between different factors or compare the prevalence and impact of specific types of events between settings.

### Recommendations for future research

If confirmed in larger studies, potential implications of these preliminary findings could include the provision of trauma-informed services for individuals with psychotic disorders and policies targeted at reducing exposure to trauma (e.g. child protection interventions), to reduce the incidence of psychosis. International Research Programme on Psychoses in Diverse Settings II (INTREPID II),^45^ which is the next phase of this study, will attempt to replicate these findings with larger samples. Future research must capture the timing of life events to establish the temporal order of these relative to the onset of psychosis, as well as the relative importance of recent events and earlier exposure. The local validity of the HTQ to capture the most relevant events to each setting should also be explored, and researchers should consider adapting these measures as needed. With larger samples, it should also be possible to investigate the impact of specific types of events, and interactions between exposure to life events and other forms of disadvantage as well as protective factors, to better understand the causes of psychosis and identify opportunities for intervention. Finally, protective factors such as social support that may buffer the effects of life events across diverse settings, potentially leading to less strong associations with psychosis in some contexts, should be investigated.

In conclusion, this exploratory study showed no overall evidence of an increase in the odds of psychosis with increasing exposure to life events, in three culturally and economically diverse countries of the Global South; however, there was some evidence of variation across settings. Further studies with larger samples should attempt to replicate these findings.

## Data Availability

Data is available upon reasonable request to C.M. (craig.morgan@kcl.ac.uk).

## Supplementary material

For supplementary material accompanying this paper visit http://doi.org/10.1192/bjo.2022.562. click here to view supplementary material
