# Supplementary material for: Life events and psychosis: case–control study from India, Nigeria, and Trinidad and Tobago
Source: BJPsych Open. 2022 Sep 16;8(5):e168. doi: 10.1192/bjo.2022.562 (PMC9534879; doi:10.1192/bjo.2022.562)
Supplement: Supplementary file 1 [file S2056472422005622sup001.docx]

SUPPLEMENTARY MATERIAL

Harvard Trauma Questionnaire

|  |  | No event | Witnessed | Experienced |
| --- | --- | --- | --- | --- |
| 1. | Combat situation |  |  |  |
| 2. | Lack of food and water |  |  |  |
| 3. | Lack of shelter |  |  |  |
| 4. | Being close to death |  |  |  |
| 5. | Illness without access to medical care |  |  |  |
| 6. | Forced separation from family members |  |  |  |
| 7. | Unnatural death of family/friend |  |  |  |
| 8. | Lost or kidnapped |  |  |  |
| 9. | Torture |  |  |  |
| 10. | Murder of family/friend |  |  |  |
| 11. | Serious injury |  |  |  |
| 12. | Imprisonment |  |  |  |
| 13. | Murder of strangers |  |  |  |
| 14. | Sexual abuse or rape |  |  |  |
| 15. | Brainwashing |  |  |  |
| 16. | Forced isolation from others |  |  |  |
| 17. | Other situations that was very frightening |  |  |  |
